# Cyclodextrin Cationic Polymer-Based Nanoassemblies to Manage Inflammation by Intra-Articular Delivery Strategies

**DOI:** 10.3390/nano10091712

**Published:** 2020-08-29

**Authors:** Annalaura Cordaro, Roberto Zagami, Milo Malanga, Jagadeesh Kumar Venkatesan, Carmen Alvarez-Lorenzo, Magali Cucchiarini, Anna Piperno, Antonino Mazzaglia

**Affiliations:** 1 CNR-ISMN, Istituto per lo Studio dei Materiali Nanostrutturati, V. le F. Stagno d’Alcontres 31, 98166 Messina, Italy; annalaura.cordaro@ismn.cnr.it (A.C.); roberto.zagami@ismn.cnr.it (R.Z.); 2 Dipartimento di Scienze Chimiche, Biologiche, Farmaceutiche ed Ambientali, Università di Messina, V. le F. Stagno d’Alcontres 31, 98166 Messina, Italy; 3 CycloLab, Illatos út 7, H-1097 Budapest, Hungary; malanga@cyclolab.hu; 4 Center of Experimental Orthopaedics, Saarland University Medical Center, Kirrbergerstr. Bldg 37, D-66421 Homburg/Saar, Germany; jegadish.venki@gmail.com (J.K.V.); mmcucchiarini@hotmail.com (M.C.); 5 Departamento de Farmacología, Farmacia y Tecnología Farmacéutica, I+DFarma (GI-1645), Facultad de Farmacia and Health Research Institute of Santiago de Compostela (IDIS), Universidade de Santiago de Compostela, 15872 Santiago de Compostela, Spain; carmen.alvarez.lorenzo@usc.es

**Keywords:** polymeric cyclodextrins, IL-1β, human marrow-derived mesenchymal stromal cells, rhodamine

## Abstract

Injectable nanobioplatforms capable of locally fighting the inflammation in osteoarticular diseases, by reducing the number of administrations and prolonging the therapeutic effect is highly challenging. β-Cyclodextrin cationic polymers are promising cartilage-penetrating candidates by intra-articular injection due to the high biocompatibility and ability to entrap multiple therapeutic and diagnostic agents, thus monitoring and mitigating inflammation. In this study, nanoassemblies based on poly-β-amino-cyclodextrin (PolyCD) loaded with the non-steroidal anti-inflammatory drug diclofenac (DCF) and linked by supramolecular interactions with a fluorescent probe (adamantanyl-Rhodamine conjugate, Ada-Rhod) were developed to manage inflammation in osteoarticular diseases. PolyCD@Ada-Rhod/DCF supramolecular nanoassemblies were characterized by complementary spectroscopic techniques including UV-Vis, steady-state and time-resolved fluorescence, DLS and ζ-potential measurement. Stability and DCF release kinetics were investigated in medium mimicking the physiological conditions to ensure control over time and efficacy. Biological experiments evidenced the efficient cellular internalization of PolyCD@Ada-Rhod/DCF (within two hours) without significant cytotoxicity in primary human bone marrow-derived mesenchymal stromal cells (hMSCs). Finally, polyCD@Ada-Rhod/DCF significantly suppressed IL-1β production in hMSCs, revealing the anti-inflammatory properties of these nanoassemblies. With these premises, this study might open novel routes to exploit original CD-based nanobiomaterials for the treatment of osteoarticular diseases.

## 1. Introduction

Osteoarthritis (OA) is a prevalent, chronic and severe degenerative disease that affects about 50% of the over-sixty population [1]. OA is characterized by alterations in the whole joint (articular cartilage degradation, bone remodeling and synovial inflammation) that lead to joint instability, failure, intermittent pain and swelling [2]. While various surgical and pharmacological treatments are available in the clinics to control the progression of OA, none of them are able to reproduce the original hyaline articular cartilage in affected patients [3,4]. Even the efficacy and/or safety of already approved drugs and formulations, such as corticosteroids and hyaluronic acid (HA) dispersions, are debated for different reasons: i) the time of residence of free drugs in the joint upon intra-articular administration is inadequate because of adverse pharmacokinetics that behave with rapid lymphatic drainage and physiological turnover of the synovial fluids and ii) the diffusion of drug trough cartilage could be slower than its clearance because of the high density of anionic extracellular matrix and small pore size (≅15 nm), thus free drugs could not achieve the therapeutic concentrations in the target site. To encompass the rapid clearance of free drug (from hours or few days to weeks) a plethora of engineered biomaterials were proposed [5]. However, sustained intra-articular delivery strategies by means of drug conjugated or entrapped in hydrogels have some limitations such as (i) the chemical modification could inactivate the drug and (ii) the delayed release of a small drug is generally achieved by increasing the crosslinking of the polymer, which could not flow through a syringe and finally not match the mechanical features of the joint [6]. To contrast the short therapeutic time, decreasing the frequency of administration, thus optimizing the cartilage penetration, more injectable nanoformulations based on cationic polyelectrolyte needed to be proposed for intra-articular delivery [7].

Therapeutic polymers are excellent candidates to get suitable nanosize-drug delivery systems with the successful features for OA treatment [8]. Cyclodextrins (CDs) are cyclic oligosaccharides capable of encapsulating guest molecules within their hydrophobic cavity via non covalent interactions. The sequestration of the guest (or part of it) inside the cavity usually improves its physicochemical properties, meanwhile increasing its solubility and protecting it from the aqueous medium (degradative enzymes, oxidants, etc.) [9]. CD polymers show additional properties with respect to their monomeric counterpart. CD units have been copolymerized to different functionalities, conjugated on the side chains or cross-linked in the backbone of polymers [10,11,12] forming multifunctional nanoconstructs for effective drug and gene delivery in vitro and in vivo [13,14]. In particular, nanocarriers based on nonionic branched CD polymers such as CD polymers self-assemblies [15], CD associative arrangements [16,17] and CD nanosponges [18,19] have been widely utilized as versatile tools for hosting drugs (i.e., anticancers, antituberculars, antimalarials, phototherapeutics, etc.) within their network and modulating their release in vitro [20,21,22,23,24]. Anionic branched CD polymers have been proposed as components of drug eluting systems [25] or fibers for stent coatings [26,27]. In this scenario, branched cationic CD polymers have been designed for several applications. They increase the permeability of drugs to biological membranes [28], easily self-assemble into optimized nanocontainers for efficient intracellular delivery [29], form nanoemulsions for oral delivery [30], or systems for targeting antimicrobial effects in biofilms [31].

Diclofenac (DCF) is one of the most widely prescribed NSAIDs (non-steroidal inflammatory drug) for its analgesic and anti-inflammatory properties. Unfortunately, similarly to other NSAIDs, DCF use is associated with some gastrointestinal (GI) side effects, including ulceration and hemorrhage. In order to mitigate such side effects, DCF has been successfully administered by controlled release systems based on CDs and their supramolecular assemblies [32,33]. The inclusion of DCF inside the CD cavity helps reducing its GI mucosal toxicity and improves the solubility of the drug, enhancing its bioavailability in the site of action and anti-inflammatory effect. The interaction between DCF and CD molecules or CD oligomers by formation of inclusion complexes has already been studied [34,35,36,37,38]. However, the stability of these complexes could be not sufficient for parental/intra-articular administration [39], and strategies using CD nanomaterials might be required to increase the bioavailability and stability of the nanocomplexes [40]. In OA treatment DCF is often used by oral administration to relieve pain and inflammation, however, the topical route is preferred due to fewer systemic side effects with comparable efficacy [41]. Furthermore, it is challenging to track the effectiveness of action of therapeutic nanoparticles in osteoarticular disorders [42]. Among the ongoing research on supramolecular self-assemblies based on CD for controlled drug/gene delivery in osteoarticular regeneration [43,44], here we develop a nanoconstruct based on branched cationic β-CD (Poly-β-amino-cyclodextrin, PolyCD) [45] entrapping DCF and anchoring a probe fluorescent (adamantanyl-Rhodamine conjugate, Ada-Rhod) [46] by a supramolecular interaction. Taking advantage of the high affinity of adamantane unit for CD cavities and of the lipophilic feature of Ada-Rhod [47], the proposed PolyCD@Ada-Rhod/DCF nanoassembly has the potentiality to become theranostic. In this study the presence of Ada-Rhod as a doping agent ([CD]:[Ada-Rhod] ≅ 33:1 molar ratio) was exploited to study the cellular uptake of the nanoassembly. The biocompatibility of the system was assayed on human bone marrow-derived mesenchymal stromal cells (hMSCs) and the decrease of intrinsic levels of IL-1β production in hMSCs was detected to evaluate the protective activities against proinflammatory responses.

## 2. Materials and Methods

### 2.1. Materials

Poly-β-amino-cyclodextrin (PolyCD, Average MW = 25 kDa, CD content 70%) was synthesized at CycloLab (Budapest, Hungary) by cross-linking ad-hoc derivatized β-CD monomer with epichlorohydrin as already reported [45]. A colorimetric Kaiser test [48,49] was performed to spectroscopically establish the quantity of amino groups present in PolyCD, which was estimated to be 0.21 mmol/g (see SI (Supplementary Information)). Adamantanyl-Rhodamine conjugate (Ada-Rhod, MW = 735.5 g/mol) was synthesized as previously reported [46]. Diclofenac sodium salt (DCF, MW = 318.13 g/mol) and all the solvents (analytical grade) were purchased from Sigma-Aldrich (Milano, Italy). All the dispersions used for nanoassemblies preparation and spectroscopic characterizations were prepared in ultrapure water (Fresenius Kabi Italia) or in 10 mM phosphate buffer containing NaCl (137 mM) and KCl (2.7 mM) at pH 7.4 (PBS) at room temperature (r.t. ≅ 25 °C). pH measurements were obtained using an 827 pH Lab pHmeter—Metrohm.

### 2.2. Nanoassemblies Preparation

#### 2.2.1. Preparation of PolyCD@Ada-Rhod

The complex was prepared at [CD]:[Ada-Rhod] ≅ 33:1 molar ratio (0.017 µmol of Ada-Rhod/mg of PolyCD) with [CD] equal to a molar concentration of repetitive units in PolyCD (see SI). Briefly, PolyCD was dissolved in ultrapure water (44 mg/4.4 mL) and sonicated in an ultrasonic bath (10 min). A thin Ada-Rhod organic film (0.6 mg) was prepared by slow evaporation of a dichloromethane (DCM) solution and this latter was hydrated with the previously prepared polymer solution (heated at 50 °C), followed by sonication in ultrasonic bath (1 h 30 min). The pink aqueous phase was collected and analyzed, whereas the residual Ada-Rhod film was separated by slight centrifugation and used to determine Ada-Rhod actual loading into the complex (by difference of the weighted amounts of Ada-Rhod initially present in organic film and the residual film after hydration).

#### 2.2.2. Preparation of PolyCD@Ada-Rhod/DCF

An organic film of DCF (17 mg at [CD]:[DCF] 1:1 molar ratio) previously prepared by slow evaporation of an acetone solution was hydrated with an aqueous solution of PolyCD or PolyCD@Ada-Rhod (92.3 mg/10 mL) and sonicated for 20 min.

All the samples were freeze-dried and then reconstituted in aqueous medium. After freeze-drying, recovery yield was calculated considering the final recovered amount of product (mg) vs. the initial weighed amount of each component.

### 2.3. Loading and Entrapment Efficiency

Both Ada-Rhod or DCF actual loading (AL%), theoretical loading (TL%) and entrapment efficiency percentages (EE%) were evaluated by UV/Vis spectroscopy using the following Equations:

(1) $$\mathrm{AL}\;\left(\%\right)=\frac{\mathrm{amount}\;\mathrm{of}\;\mathrm{Ada}-\mathrm{Rhod}\;\mathrm{or}\;\mathrm{DCF}\;\mathrm{into}\;\mathrm{the}\;\mathrm{nanoassembly}}{\mathrm{weight}\;\mathrm{of}\;\mathrm{nanoassembly}}\times 100$$

(2) $$\mathrm{TL}\;\left(\%\right)=\frac{\mathrm{amount}\;\mathrm{ofAda}-\mathrm{Rhod}\;\mathrm{or}\;\mathrm{DCF}\;\mathrm{initially}\;\mathrm{added}\;\mathrm{to}\;\mathrm{formulation}}{\mathrm{weight}\;\mathrm{of}\;\mathrm{nanoassembly}}\times 100$$

(3) $$\mathrm{EE}\;\left(\%\right)=\frac{\mathrm{amount}\;\mathrm{of}\;\mathrm{Ada}-\mathrm{Rhod}\;\mathrm{or}\;\mathrm{DCF}\;\mathrm{in}\;\mathrm{nanoassembly}}{\;\mathrm{amount}\;\mathrm{of}\;\mathrm{Ada}-\mathrm{Rhod}\;\mathrm{or}\;\mathrm{DCF}\;\mathrm{initially}\;\mathrm{added}\;\mathrm{to}\;\mathrm{formulation}}\times 100$$

The amount of Ada-Rhod loaded into the system was calculated by redissolving the residual film from the complexation reaction in DCM and measuring its absorption intensity. A Lambert and Beer calibration curve for Ada-Rhod in DCM was performed in the concentration range 25–200 µM (ε = 877.8 ± 10 M^−1^ cm^−1^; see Figure S1).

DCF actual loading inside PolyCD@Ada-Rhod/DCF system and EE% were evaluated by UV/Vis by means of difference by DCF initially added and residue in organic film after hydration. Calibration curves for free DCF were performed both in ultrapure water and PBS: the calculated molar extinction coefficients were respectively 8130 ± 225 M^−1^ cm^−1^ (DCF free in water) and 9700 ± 767 M^−1^ cm^−1^ (DCF free in PBS; see Figure S2).

### 2.4. UV/Vis and Steady State and Time Resolved Fluorescence Spectroscopy

UV/Vis spectra were obtained on a Agilent model 8453 diode array spectrophotometer using 1 cm path length quartz cells at T = 25 °C by using a thermostatic bath. Steady-state fluorescence measurements were performed on a Jasco model FP-750 spectrofluorimeter by using a 0.5 or 1 cm path length quartz cells. Time resolved fluorescence emission measurements were performed on a Jobin Yvon-Spex Fluoromax 4 spectrofluorimeter using time-correlated single-photon counting technique and a NanoLED (λ = 390 nm) as the excitation source, as already reported [50,51].

### 2.5. Job Plot and Characterization of the PolyCD/DCF Complex in Solution

Job’s plot experiments were performed by two equimolar stock solutions of PolyCD and DCF ([CD] = [DCF] = 1 mM) both by mixing them in ultrapure water and maintaining the total volume and concentration constant ([CD] + [DCF] = 100 µM) where [CD] is the molar concentration of repetitive unit in PolyCD. Accordingly, the molar fraction (χ) was changed from 0.1 to 1 and measuring the corresponding absorbance by UV/Vis at T = 25 °C. Plots show the χ vs. ΔA/A_0_ × [DCF], where ΔA is the difference between absorbance values at maxima in the presence (A) and in the absence (A_0_) of PolyCD respectively vs. χ_DCF_ (where χ_DCF_ is the molar fraction of DCF at the investigated molar concentration [DCF]) [52,53].

The complexation of DCF in PolyCD was studied by UV/Vis titration. Different aqueous solutions of free DCF and DCF with different amounts of PolyCD ([DCF] = 100 µM and [CD] varying in the range 0–150 μM) were prepared in sealed vials by adding aliquots of PolyCD to aqueous solutions of DCF, homogenized by slight sonication (10 min) and thermally equilibrated at T = 25 °C. The dispersions were analyzed by UV/Vis as described and the plot of 1/[A − A_0_] as a function of 1/[CD] was reported, where A and A_0_ are the absorbance of DCF in the presence and in the absence of PolyCD measured at λ_max_ of complex absorbance.

### 2.6. Size and ζ-Potential Measurements

Hydrodynamic diameter (D_H_) or size, width of distribution (polydispersity index, PDI) and ζ-potential of the PolyCD-based nanoassemblies were determined by photon correlation spectroscopy (PCS) by a Zetasizer Nano ZS (Malvern Instrument, Malvern, U.K.) at 25 °C in ultrapure water. The measurements were carried out at 173° angle vs. the incident beam at 25 ± 1 °C for each aqueous dispersion. The deconvolution of the correlation curve to an intensity size distribution was obtained by using a non-negative least-squares algorithm. The ζ-potential values were measured using a Zetasizer Nano ZS Malvern Instrument equipped with a He−Ne laser at a power P = 4.0 mW and λ = 633 nm. The results are reported as the mean of three separate measurements on three different batches ± the standard deviation (SD).

### 2.7. Stability Studies

Stability studies were carried out by dissolving PolyCD@Ada-Rhod/DCF (0.5 mg/mL) in different biological media: (i) ultrapure water, (ii) 0.9 wt % NaCl aqueous solution, (iii) PBS at pH 7.4. All the solutions were kept under stirring (T = 25 °C) along 14 days and analyzed by UV/Vis and DLS at r.t. in triplicate. ζ-Potential was measured along 2 weeks on the dispersions prepared in ultrapure water and stored at 25 °C.

### 2.8. Release Studies

Release profile of DCF from PolyCD@Ada-Rhod/DCF nanoassembly was evaluated in PBS at pH 7.4 by a dialysis method. PolyCD@Ada-Rhod/DCF (10 mg) in PBS (1 mL) were put into a dialysis tube (Spectra/Por^®^ dialysis bags, MWCO 3.5 kDa) and immersed into 10 mL of PBS (sink condition) under continuous stirring (250 rpm) at 37 ± 0.5 °C. At fixed times, 1 mL of release medium was withdrawn and replaced with an equal volume of fresh aqueous solution of PBS. The amount of DCF released was evaluated by UV/Vis spectroscopy (at λ = 276 nm) and was expressed as percentage ratio between the weight of released DCF and the total amount of entrapped drug. The kinetic analysis was carried by three models proposed in the literature such as Higuchi, Baker–Lonsdale and the first order process (see infra and SI) [53,54].

### 2.9. Biological Studies

#### 2.9.1. Materials

All reagents were from Sigma (Munich, Germany) unless otherwise indicated. Recombinant FGF-2 (rFGF-2) was purchased at R&D Systems (Wiesbaden-Nordenstadt, Germany). The Cell Proliferation Reagent WST-1 and the Cytotoxicity Detection KitPLUS (LDH) were obtained at Roche Applied Science (Mannheim, Germany). The Human IL-1β and TNF-α enzyme-linked immunosorbent assays (ELISAs; Human IL-1β Quantikine ELISA, TNF-α Quantikine ELISA) were from R&D Systems. 

#### 2.9.2. Cell Culture

Bone marrow aspirates (15 mL) were obtained from distal femurs of patients undergoing total knee arthroplasty (*n* = 8, age 68–74 years). The study was approved by the Ethics Committee of the Saarland Physicians Council. All procedures were in accordance with the Helsinki Declaration and all patients provided informed consent before inclusion in the study. Bone marrow-derived human mesenchymal stromal cells (hMSCs) were isolated according to standard protocols [55,56] by washing and centrifuging the aspirates in Dulbecco’s modified Eagle’s medium (DMEM). The cell pellet was resuspended in red blood cell lysing buffer (Sigma) and DMEM (1:1). The mixture was washed, pelleted and resuspended in DMEM with 10% fetal bovine serum, 100 U/mL penicillin and 100 µL/mL streptomycin (growth medium). The cells were plated in T75 flasks and kept at 37 °C under 5% CO_2_ overnight. The medium was then removed and replaced by growth medium with recombinant FGF-2 (1 ng/mL), with medium exchanged every 2–3 days. Proliferating cells were replated when reaching an 85% density and hMSCs were further used at no more than passage 1–2. Cell studies were carried out by adding PolyCD-based nanoassemblies (2 mg/mL, [Ada-Rhod] = 32 µM, [DCF] = 944 µM).

#### 2.9.3. Detection of Live Fluorescence

hMSCs were seeded in 24-well plates (2 × 10^4^ cells/well) with growth medium for 12 h at 37 °C under 5% CO_2_. PolyCD-based nanoassemblies were then directly added to the cultures and live fluorescence was monitored in the samples by fluorescent microscopy using a rhodamine filter set (568 nm; Olympus CKX41; Hamburg, Germany) [55,57].

#### 2.9.4. Cell Proliferation and Viability

hMSCs were seeded in 24-well plates (2 × 10^4^ cells/well) with growth medium for 12 h at 37 °C under 5% CO_2_ prior to direct addition of the PolyCD-based nanoassemblies to the cultures. Cell proliferation was evaluated using the Cell Proliferation Reagent WST-1 according to the manufacturer’s recommendations [55,56,57]. Cell viability was determined with the Cytotoxicity Detection KitPLUS (LDH) in the supernatants of culture by assessing the absorbance at 450 nm on a GENios spectrophotometer/fluorometer (Tecan, Crailsheim, Germany). Cytotoxicity was calculated as follows [57]:

cell viability (%) = (experimental value − low control)/(high control − low control) × 100(4)

#### 2.9.5. Inflammatory Responses

hMSCs were seeded in 24-well plates (2 × 10^4^ cells/well) with growth medium for 12 h at 37 °C under 5% CO_2_ prior to direct addition of the PolyCD-based nanoassemblies to the cultures. Inflammatory responses were monitored by measuring the production levels of IL-1β and TNF-α in the supernatants of culture by respective ELISAs on a GENios spectrophotometer/fluorometer.

#### 2.9.6. Statistical Analysis

All tests were performed in triplicate in three independent experiments. Data are expressed as mean ± standard deviation (SD) of separate experiments. The *t*-test was employed where appropriate, with *p* < 0.05 considered statistically significant.

## 3. Results and Discussion

### 3.1. Nanoassemblies Preparation 

**Scheme 1:**
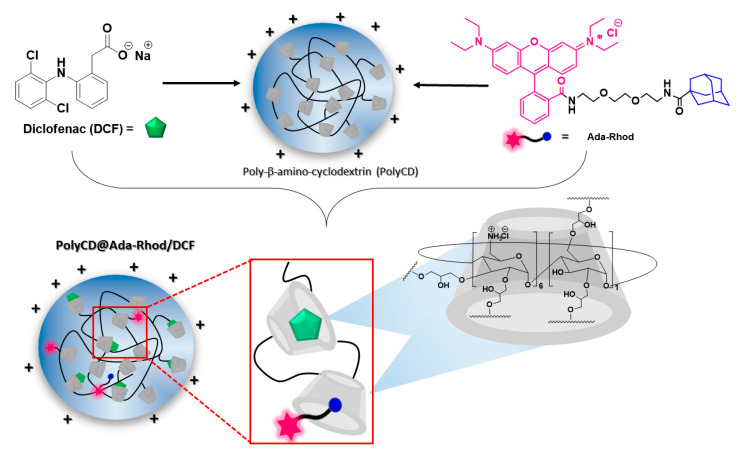
Sketched view of nanoassemblies preparation (PolyCD@Ada-Rhod/DCF).

Fluorescent cationic nanoassemblies entrapping DCF (PolyCD@Ada-Rhod/DCF) were prepared by hydration of Ada-Rhod organic film and the recovered PolyCD@Ada-Rhod was used for the following hydration of DCF organic film. Concentration of CD repetitive units was used in slight excess vs. [DCF], thus to achieve a complete drug entrapment. Scheme 1 summarizes nanoassemblies formation by molecular components.

Nanoassemblies were obtained with high Ada-Rhod and DCF entrapment efficiency (≅92% and 100%, respectively). Ada-Rhod residual film was used to determine Ada-Rhod loading (see experimental). No residual of DCF was found in the dispersions of PolyCD@Ada-Rhod/DCF, confirming the complete entrapment. Moreover, it was observed that the recovery yield for all systems is 80%, probably because of the presence of a little water percentage in the starting cyclodextrin polymer, due to its highly hygroscopic nature. Properties of nanoassemblies are reported in Table 1.

DLS analysis of PolyCD and PolyCD@AdaRhod/DCF nanoassemblies (Table 1) shows a size distribution centered at a hydrodinamic diameter (D_H_) of about 250 nm for the main population and a ζ-potential of about + 20 mV, due to the positive charges of the amino groups of the polymer network (see Figure S3). Surprisingly PolyCD@AdaRhod showed a size that is about two-fold vs. the analogue with DCF, suggesting a different rearragment vs. the nanossemblies entrapping both Ada-Rhod and DCF.

### 3.2. Interaction Studies and Complexes Formation

**Figure 1:**
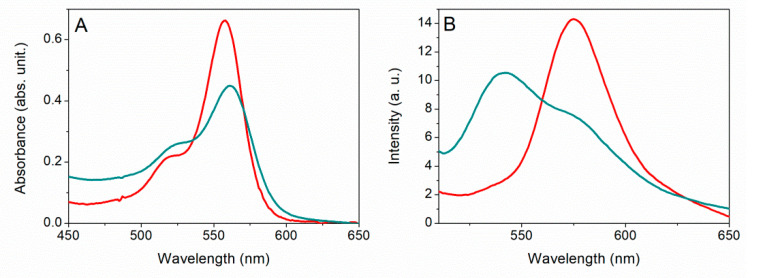
UV/Vis spectra (**A**) and steady steady state emission spectra (**B**) of free Ada-Rhod in DCM (red trace) and PolyCD@Ada-Rhod (blue cyan trace) in ultrapure water, pH = 4. (**A**): 44 mg/mL, [Ada-Rhod] = 713 µM, pH = 4, d = 1 cm and scattering subtracted in the spectrum of the PolyCD@Ada-Rhod. (**B**): 0.5 mg/mL, [Ada-Rhod] = 8 µM, λ_exc_ = 480 nm.

The interactions of PolyCD with Ada-Rhod within PolyCD@Ada-Rhod, and with both Ada-Rhod and DCF within PolyCD@Ada-Rhod/DCF were investigated by UV/Vis, steady-state and time-resolved fluorescence emission. PolyCD/DCF complex formation was studied for comparison. UV/Vis spectra and fluorescence emission of free Ada-Rhod in DCM vs. PolyCD@Ada-Rhod complex are shown in Figure 1. Ada-Rhod’s absorption profile shows a major band centered at 558 nm in DCM, which was red-shifted at 561 nm in PolyCD@Ada-Rhod. The appearance of absorption profile in water was unambiguous evidence of Ada-Rhod complexation since free Ada-Rhod was not soluble in water (Figure 1A). Steady state emission fluorescence (Figure 1B) of Ada-Rhod in DCM shows a band centered at 575 nm, whereas after the interaction with PolyCD in aqueous medium the emission profile actually split into a band, centered at 542 and a shoulder around 576 nm respectively.

This double band profile is typical of rhodamine derivatives because of the tautomeric equilibrium that occurs in water, and is strictly influenced even by slight pH changes in the aqueous microenvironments [58,59].

**Figure 2:**
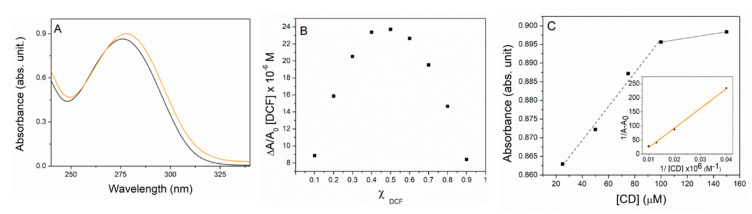
(**A**) UV/Vis spectra of free DCF (black trace) and PolyCD/DCF (orange trace) in water, [CD] = [DCF] = 100 µM; (**B**) Job’s plot for the complexation of DCF with PolyCD from UV/Vis measurements in ultrapure water (see Experimental section); and (**C**) UV/Vis spectral changes of DCF vs. CD concentration (dashed black traces) and plot of (1/ΔA) vs. 1/[CD] (inset, orange trace, R^2^ = 0.997) in ultrapure water, [DCF] = 100 μM, [CD] = 25–150 μM; d = 1 cm; T = 25 °C.

The interaction of PolyCD with DCF was firstly studied by UV/Vis spectroscopy. The complexation of the drug into PolyCD was obtained by simple mixing of aqueous solutions of DCF and PolyCD as reported in experimental method. The incorporation of DCF into the complex was evident from the UV/Vis spectrum (Figure 2A) that displays a band centered at 276 nm for free DCF (black trace) and 278 nm for the complex in water (orange trace). Furthermore, a slight hyperchromic effect was observed upon complexation. For comparison, a UV/Vis spectrum was recorded on PolyCD/DCF complex obtained by solvent evaporation technique (hydration of organic film and sonication) as reported in the experimental method and the same effect was observed, thus confirming the interaction (Figure S4). Probably the very slight hypercromicity and shift are due to the excellent dispersibility of both components in water. The stoichiometry of the complex was determined by the continuous variation method [60]. The shape of Job’s plot (ΔA/A_0_ × [DCF] vs. χ DCF) was highly symmetrical, showing a maximum value at χ DCF = 0.5 pointing out a formation of complex with prevalent 1:1 stoichiometry (Figure 2B).

Furthermore, the formation of complex was confirmed by increase of absorbance measured vs. CD concentration in the range 25–150 µM. This plot releaves a bimodal behaviour, with a linear increase up to 100 µM and afterwards a pseudo plateau (Figure 2C). In the first linear portion a A_L_ type diagram with a slope less of a unit was observed. At higher host concentrations complexes with a higher order and undefined stochiometry could occur. The apparent binding constant for the 1:1 complex can be evaluated by using the Benesi–Hildebrand Equation [61].

(5) $$\frac{1}{A-{\;A}_{0}}=\;\frac{1}{K_{b}\;\times \left(A_{\max\;}-{\;A}_{0}\right)\times \left[\mathrm{CD}\right]}+\;\frac{1}{A_{\max\;}-{\;A}_{0}}$$

where A is the absorbance at maximum of the PolyCD/DCF complex, A_0_ is the absorbance of DCF in the absence of PolyCD, [CD] is the PolyCD concentration in CD units, A_max_ is the absorbance at [CD] _max_ (100 µM) and K_b_ is the apparent binding constant. The apparent binding constant was estimated from the slope /A_max_ − A_0_ (plot of 1/(A − A_0_) *vs.* 1/[CD]) and is found to be 4.1 × 10^3^ M^−1^ (log K_b_ ≅ 3.60; inset of Figure 2C). This value agrees with data found for complexation of DCF in cationic CD cross-linked oligomers (log K_b_ ≅ 3.47) [38]. 

**Figure 3:**
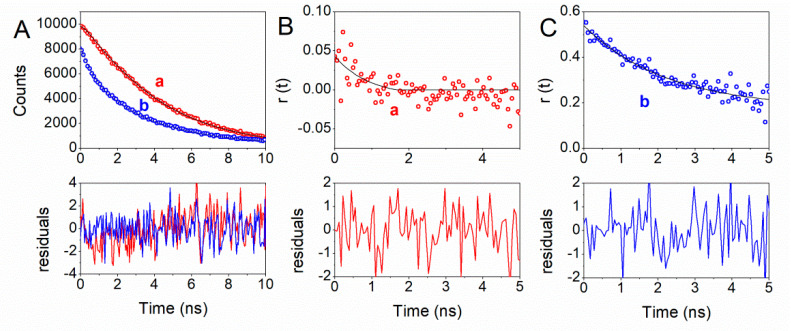
Fluorescence time decay (**A**) and time-resolved fluorescence anisotropy (**B**,**C**) of a) Ada-Rhod 100 µM in DCM and b) PolyCD@Ada-Rhod/DCF (0.5 mg/mL, [Ada-Rhod] = 8 μM, [DCF] = 236 µM) in ultrapure water at room temperature (r.t.).

Fluorescence time-decays of Ada-Rhod free and within nanoassemblies were fitted by one and three exponential profiles respectively, estimating three different fluorescence lifetimes when the probe is complexed into the polymeric structure (Table 2 and Figure 3).

The time fluorescence decay (Figure 3) and correspondent fluorescent lifetimes (Table 2) pointed out that free Ada-Rhod in DCM was present mostly as a monomer (τ_1_ = 3.6 ns) [62,63,64]. When analyzing PolyCD@Ada-Rhod/DCF nanoassemblies in ultrapure water, three lifetimes were observed and one of these (τ_2_ = 2.4 ns) was ascribable to Ada-Rhod species entrapped within the polymer chains in the monomeric form [46,65]. This could also explain the rotational correlation time after interaction of Ada-Rhod within PolyCD@Ada-Rhod/DCF (0.8 ns in free Ada-Rhod vs. 2.2 ns in the nanoassembly), suggesting that the probe is effectively incorporated into the structure, but still maintains a certain freedom to rotate upon itself [51]. For what concerns the shorter lifetimes (0.4 ns in the PolyCD@Ada-Rhod/DCF) it could be supposedly due to self-oligomers formation of Ada-Rhod, likely generated by self π–π stacking or other aggregation phenomena that lead to fluorescence quenching. Finally, the longer ones (τ_3_ = 5.8 ns) were tentatively ascribed to species of Ada-Rhod interacting more closely to CD cavities (i.e., inclusion of Ada portion).

Overall, our investigations indicated that both DCF and Ada-Rhod interact with CD cavities. Host–guest complexation of Ada-Rhod takes advantage of the high affinity of adamantane portion for CD cavities (K_b_ = 5 × 10^4^ M^−1^) [66], and this interaction is stronger with respect to DCF with a CD cavity: indeed no Ada-Rhod displacement was observed even if DCF was used in excess vs. Ada-Rhod. The formation of larger aggregates in PolyCD@Ada-Rhod rather than PolyCD@Ada-Rhod/DCF is an experimental evidence that was already observed in others nanoassemblies based on polymeric systems functionalized with rhodamine [67]. We suppose that upon inclusion of the adamantane unit into CD cavity, the rhodamine residue of Ada-Rhod is located in a more hydrophilic environment, likely outside the CD cavity and in the proximity of the CD rims. Moreover, the formation of supramolecular self-oligomers of Ada-Rhod with shorter fluorescence lifetimes (0.4 ns with amplitude of 23%) could occur. These arrangements could allow in average an increase of D_H_ (see Table 1) in PolyCD@Ada-Rhod. On the other hand, the decrease of D_H_ and ζ-potential values in PolyCD@Ada-Rhod/DCF could be tentatively ascribed to the formation of more compacted and smaller nanoassemblies due to an increased colloidal stability originated from the significant interactions of DCF with both CD cavity and cationic external CD rims, as clearly indicated by UV-Vis, DLS and ζ-potential results.

### 3.3. Stability and Release Studies

**Figure 4:**
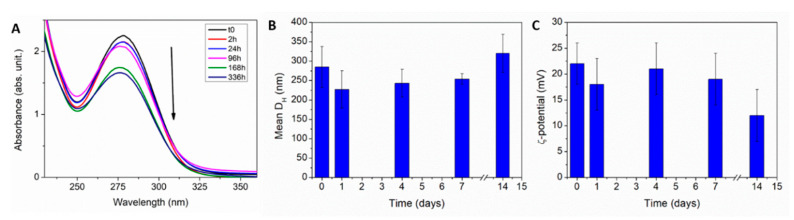
UV/Vis spectra (**A**), mean D_H_ (main populations only) (**B**) and ξ-potential (**C**) vs. time of PolyCD@Ada-Rhod/DCF nanoassemblies in ultrapure water (0.5 mg/mL, [Ada-Rhod] = 8 µM, [DCF] = 236 µM). Data were acquired at t = 0, 1, 4, 7 and 14 days. Nanoassemblies dispersions were stored at 25 °C under stirring along the experimental time.

In order to investigate if the aggregation properties of the nanoassemblies are influenced by a dispersing medium, we carried out stability studies vs. time in ultrapure water and in biologically relevant media by DLS and UV/Vis. For freshly prepared solutions at t = 0, the UV/Vis profile of DCF within PolyCD@Ada-Rhod/DCF showed the band centered at 278 nm. The changes of absorption spectra, D_H_ and ζ-potential vs. time for PolyCD@Ada-Rhod/DCF nanoassemblies in ultrapure water are shown in Figure 4. Within two weeks, the absorbance of the band at 278 nm decreased about 30% with respect to the freshly prepared dispersion. Size of the nanoassemblies was monitored for up to 2 weeks. At t = 0, all the samples exhibited a size distribution with a mean diameter roughly of 250 nm, which remains approximately constant for the first 7 days. The ζ-potential values vs. time were measured in ultrapure water at r.t. With a starting value at t = 0 around 20 mV, a significant decrease was observed from the 7th day on. However, the sharply positive surface charge is advantageous for a rapid cellular uptake of the nanoassemblies from the target cells, which should be completed far before one week from the local administration.

As far as it concerns the other biological media (PBS and NaCl 0.9 wt %), an immediate increase in size was observable after the first day (see Figures S5 and S6), suggesting that the aggregation properties of the nanoassemblies can be influenced by the medium ionic strength. After 2 weeks, the size significantly increased for all the dispersions in all media.

In conclusion, it is possible to assess that PolyCD@Ada-Rhod/DCF nanoassemblies were stable in aqueous medium at 25 °C up to 7 days. On the contrary, it is advisable to administer the saline injectable formulation within the first day after reconstitution, for maintaining a high concentration of DCF loaded in the nanoformulation. This expedient could allow in view of the potential application of these nanosystems to further prolong and sustain their performance in the joint [68,69,70].

**Figure 5:**
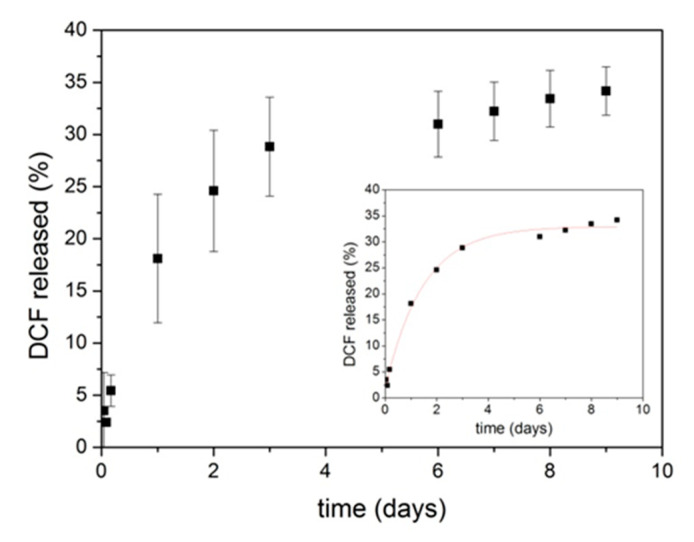
Release profile of DCF from PolyCD@Ada-Rhod/DCF nanoassemblies in PBS (pH = 7.4) at 37 °C (Spectra/Por® dialysis bags, MWCO 3.5 kDa). Data are reported as the mean three independent experiments ± SD. In the inset experimental data are best-fitted first order kinetic model.

Kinetic release profiles of DCF from PolyCD@Ada-Rhod/DCF were evaluated in PBS at 37 °C up to 10 days (Figure 5). Released DCF was determined using the calibration curve by UV/Vis of DCF in PBS (Figure S2B). No initial burst release was observed from the nanoassembly, with a slow and controlled release in time, leading to a final 35% of DCF in the external medium. The corresponding unreleased amount of drug in the donor compartment (60%) was determined by UV/Vis analysis. 

The kinetic analysis was carried out by three different models reported in the literature by Higuchi, Baker–Lonsdale and a simple first order process for different pharmaceutical dosage forms (i.e., semisolid and solid; see Table 3 and SI) [54,71] and potentially applicable to nanoparticulate dispersions [53,72].

By best-fit of data (inset of Figure 5) it was possible to evaluate the regression coefficient (R^2^) and release parameters (k/h^−1^). By considering a first order process, as commonly known for molecular permeation across a membrane from a stirred donor into a stirred receiving phase under quasi-stationary and sink conditions [73], the model can be represented by the following Equation:

(6) $$C_{t}={\;C}_{\infty }{\left(\;1-e^{-\mathrm{kt}}\right)}^{\;}$$

where $C_{t}\;$ and $C_{\infty }$ are the amount of drug released (in percentage) in the receiving phase at time t and at t_∞_, at the end of release process, and k is the rate constant of the process. Altogether, kinetic analysis agrees with a simple monoexponential decay that is the release of DCF from the nanoassembly is a dependent first order process, showing a plateau of drug released (≅36%) after about 10 days.

### 3.4. Biological Studies

#### 3.4.1. Effective Targeting of hMSCs with PolyCD-based Nanoassemblies

**Figure 6:**
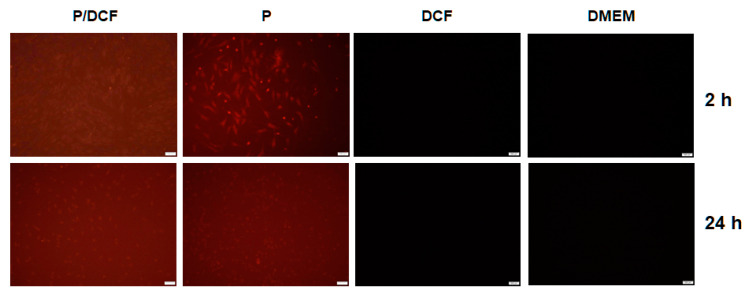
Detection of live fluorescence in human mesenchymal stromal cells (hMSCs) treated with PolyCD-based nanoassemblies (2 mg/mL). hMSCs (2 × 10^4^ cells/well in 24-well plates) were directly incubated with the compounds and live fluorescence was monitored after 2 and 24 h by fluorescent microscopy as described in the Materials and Methods (scale bars: 200 µm). Abbreviations: P/DCF: PolyCD@Ada-Rhod/DCF; P: PolyCD@Ada-Rhod. DMEM: Dulbecco’s modified Eagle’s medium.

hMSCs were treated with PolyCD-based nanoassemblies in order to examine their ability to deliver DCF. A strong fluorescent signal was detected in hMSCs receiving PolyCD@Ada-Rhod/DCF already after 2 h of incubation and for at least 24 h compared with the control conditions (positive control: PolyCD@Ada-Rhod in absence of DCF; negative controls: DCF alone, DMEM; Figure 6).

#### 3.4.2. Cell Proliferation and Viability of hMSCs Treated with the PolyCD-based Nanoassemblies

**Figure 7:**
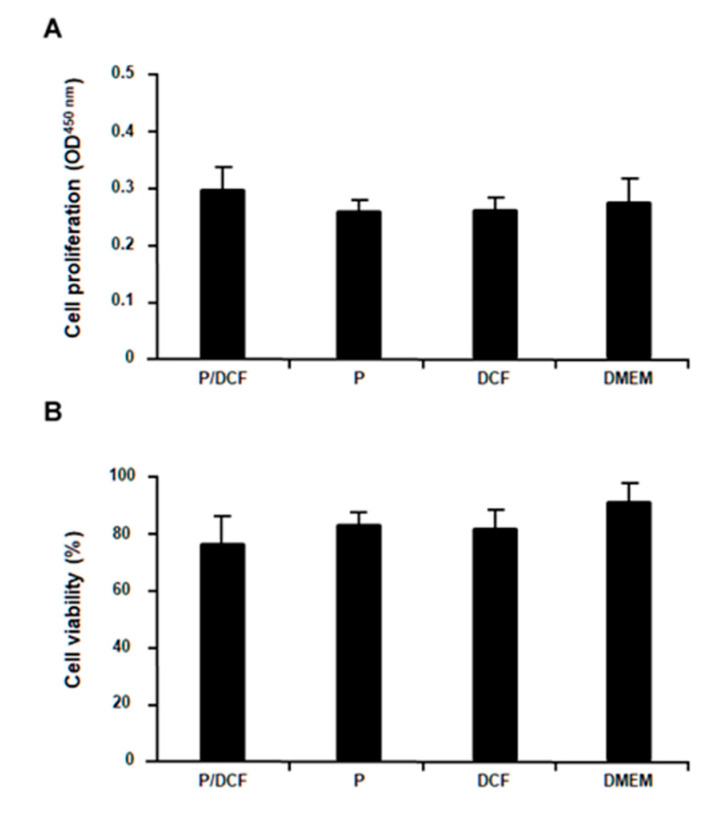
Analysis of cell proliferation and viability of hMSCs treated with the PolyCD-based nanoassemblies (2 mg/mL). hMSCs (2 × 10^4^ cells/well in 24-well plates) were directly incubated with the compounds. Cell proliferation was evaluated using the Cell Proliferation Reagent WST-1 (**A**) and cell viability with the Cytotoxicity Detection KitPLUS (LDH) (**B**) after 48 h. Abbreviations: P/DCF: PolyCD@Ada-Rhod/DCF; P: PolyCD@Ada-Rhod.

PolyCD-based nanoassemblies were next applied to the hMSCs to evaluate their potential deleterious effects on the levels of cell proliferation and viability. Importantly, no detrimental effects of the PolyCD@Ada-Rhod/DCF treatment relative to all other control conditions (PolyCD@Ada-Rhod in the absence of DCF, DCF alone and DMEM) were observed after 48 h on the cell proliferation indices (*p* ≥ 0.087; Figure 7A) or on the percent of cell viability *(p* ≥ 0.081; Figure 7B).

#### 3.4.3. Protective Effects of DCF Treatment Against Inflammation in hMSCs Treated with PolyCD-based Nanoassemblies

**Figure 8:**
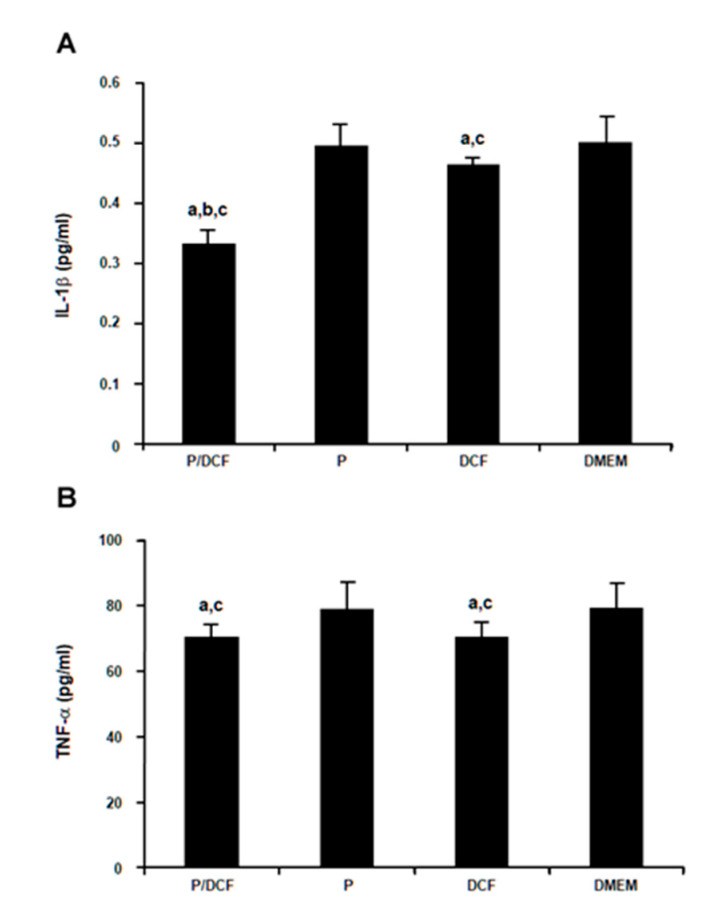
Analysis of inflammatory responses in hMSCs treated with PolyCD-based nanoassemblies (2 mg/mL). hMSCs (2 × 10^4^ cells/well in 24-well plates) were directly incubated with the compounds. Inflammatory responses (**A**: IL-1β; **B**: TNFα) were detected using IL-1β and TNF-α ELISAs, respectively, after 48 h as described in the Materials and Methods. Statistically significant compared with DMEM (a), DCF (b), and P (c). Abbreviations: P/DCF: PolyCD@Ada-Rhod/DCF; P: PolyCD@Ada-Rhod.

PolyCD@Ada-Rhod/DCF was finally provided to hMSCs to evidence possible protective activities of the system against proinflammatory responses. Remarkably, the intrinsic levels of IL-1β production in hMSCs were significantly reduced after 48 h in the presence of PolyCD@Ada-Rhod/DCF versus all other conditions (PolyCD@Ada-Rhod in absence of DCF, DCF alone, DMEM; up to 1.6-fold decrease, *p* ≤ 0.002; Figure 8A). Application of DCF alone also displayed a protective effect against IL-1β production relative to PolyCD@Ada-Rhod (without DCF) and DMEM but to a lesser extent than when provided via the PolyCD compound (up to 1.1-fold decrease, *p* ≤ 0.040; Figure 8A). The intrinsic levels of TNF-α production in hMSCs were also significantly reduced after 48 h in the presence of PolyCD@Ada-Rhod/DCF versus PolyCD@Ada-Rhod (without DCF) and DMEM (up to 1.2-fold decrease, *p* ≤ 0.003), but without significant difference relative to DCF alone (*p* = 0.188) that also had a protective effect against TNF-α production versus PolyCD@Ada-Rhod (without DCF) and DMEM (up to 1.1-fold decrease, *p* ≤ 0.001; Figure 8B).

## 4. Conclusions

In summary, the present work shows the possibility to develop fluorescently labeled PolyCD-based nanoassemblies entrapping the non-steroidal anti-inflammatory drug diclofenac (DCF) as therapeutic, anti-inflammatory nanomedicines to tackle critical pathological inflammation in OA. PolyCD was selected to take advantage of its intrinsic capability to encapsulate a wide variety of active agents and to regulate the release mechanisms by exploiting the affinity constant of the polymer for complexed guests and their hydrophilic/lipophilic features. In this study, we exploited PolyCD as a suitable theranostic platform with simultaneous loading of the therapeutic agent DCF and of the fluorescent probe Ada-Rhod. The elucidation of the PolyCD@Ada-Rhod/DCF physicochemical properties, such as size, surface charges and stability in biologically relevant media, release, etc., suggested the adequacy of PolyCD-based nanoassemblies for intra-articular delivery strategies. PolyCD-based nanoassemblies were stable in aqueous medium at 25 °C for up to 7 days and one day in saline injectable formulation after reconstitution. Release experiments evidenced a different aptitude to deliver the payloads: a retarded release without burst effect (with a plateau of released DCF (≅35%, after about 10 days) was found for DCF, whereas no trace of florescent probe (Ada-Rhod) was detected in the aqueous medium. In the particular case of the intra-articular delivery, the size and zeta potential of the polyCD@Ada-Rhod/DCF appeared suitable to develop a syringeable formulation that could sustainably release the anti-inflammatory drug for several weeks at the site of injection, allowing for a localized treatment. Importantly, the results of the biological evaluations revealed no detrimental effects of PolyCD@Ada-Rhod/DCF on cell proliferation and viability in hMSCs and a fast cellular uptake (within 2 h) in the cells that remained fluorescent until 24 h. Finally, the data demonstrated the significant anti-inflammatory potential of PolyCD@Ada-Rhod/DCF against the production of IL-1β in hMSCs in vitro, as a promising future tool to contain inflammatory pathomechanisms in OA.

## Figures and Tables

**Table 1:** Overall properties of PolyCD-based nanoassemblies: mean D_H_, polidispersity index (PDI) and ζ-potential values (ζ), loading and EE% in ultrapure water.

Sample	Medium	Mean D_H_ (nm ± SD) *^a,^* (%) *^b^*	PDI	ζ (mV ± SD)	Theoretical Loading (%)	*^c^* Actual Loading (%)	*^d^* EE (%)
PolyCD	H_2_O	268 ± 10 (97)	0.07	19 ± 6			
PolyCD@Ada-Rhod	H_2_O	498 ± 54 (85)	0.2	25 ± 5	1.28 ^(1)^	1.18 ± 0.04 ^(1)^	92.0 ± 3.3 ^(1)^
PolyCD@ AdaRhod/DCF	H_2_O	229 ± 35 (85) 29 ± 13 (12)	≤0.3	22 ± 4	1.18 ^(1)^	1.09 ± 0.05 ^(1)^	92.0 ± 3.9 ^(1)^
					15.5 ^(2)^	15.5 ^(2)^	~100 ^(2)^
	PBS pH 7.4	230 ± 24 (83) 25 ± 13 (11)	≤0.2				
	NaCl (0.9 wt %)	228 ± 21 (89) 18 ± 13 (12)	≤0.2				

*^a^* SD was calculated on three different batches. *^b^* Mean size with corresponding intensity % distribution (only main populations). *^c^* Actual loading is expressed as the amount of drug (mg) encapsulated per 100 mg of nanoassembly. *^d^* Ratio between actual and theoretical loading × 100. ^(1)^ Values are referred to Ada-Rhod; ^(2)^ Values are referred to Diclofenac; PolyCD (0.5 mg/mL) and PolyCD@Ada-Rhod/DCF (0.5 mg/mL, [Ada-Rhod] = 8 μM, [DCF] = 236 µM).

**Table 2:** Fluorescence lifetimes (τ) and rotational correlation time (θ_R_) of PolyCD@Ada-Rhod/DCF vs. Ada-Rhod.

Sample	τ_1_ ± 0.1, ns	τ_2_ ± 0.1, ns	τ_3_ ± 0.1, ns	A_1_, %	A_2_, %	A_3_, %	θ_R_ ± 0.2, ns
Ada-Rhod ^a^	3.6	--	--	100	--	--	0.8
PolyCD@ Ada-Rhod/DCF ^a^	0.4	2.4	5.8	23	34	43	2.2

^a^ Fluorescence lifetimes were measured at λ_exc_ = 390 nm: Ada-Rhod in DCM, λ_em_ = 576 nm; PolyCD@Ada-Rhod/DCF in ultrapure water, λ_em_ = 576 nm. A is the amplitude of the intensity decay. Experimental conditions: free [Ada-Rhod] = 100 µM; PolyCD@Ada-Rhod/DCF (0.5 mg/mL, [Ada-Rhod] = 8 μM, [DCF] = 236 µM).

**Table 3:** Regression coefficient (R^2^) and rate constants (k_H_ and k) of DCF release data from PolyCD@Ada-Rhod /DCF fitted according to different kinetic models.

Higuchi		Baker-Lonsdale		First order	
R^2^	k_H_ (h^−1/2^)	R^2^	k(h^−1^)	R^2^	k(h^−1^)
0.9047	12.8057 ± 0.6679	0.9241	3.3 × 10^−3^ ± 3 × 10^−4^	0.9952	0.6828 ± 0.0552

## Supplementary Materials

The following are available online at https://www.mdpi.com/2079-4991/10/9/1712/s1: 1. Estimation of CD cavities in PolyCD and amino groups content; 2. Analysis of release kinetic; Figure S1: Lambert-Beer of free Ada-Rhod in DCM; Figure S2: Lambert-Beer of free DCF in ultrapure water and PBS; Figure S3: Size (or D_H_) distribution of PolyCD (black line) and PolyCD@Ada-Rhod/DCF; Figure S4: UV/Vis of free DCF and PolyCD/DCF in ultrapure water obtained by solvent evaporation technique; Figure S5: Stability studies of PolyCD@Ada-Rhod/DCF in NaCl wt. 0.9%; Figure S6: Stability studies of PolyCD@Ada-Rhod/DCF in PBS.
