# Supplementary material for: Cyclodextrin Cationic Polymer-Based Nanoassemblies to Manage Inflammation by Intra-Articular Delivery Strategies
Source: Nanomaterials (Basel). 2020 Aug 29;10(9):1712. doi: 10.3390/nano10091712 (PMC7558260; doi:10.3390/nano10091712)
Supplement: Supplementary file 1 [file nanomaterials-10-01712-s001.pdf]

nanomaterials

Electronic Supporting Information:

Cyclodextrin Cationic Polymer-based Nanoassemblies to Manage Inflammation by Intra-articular Delivery Strategies

Annalaura Cordaro [1,2], Roberto Zagami [1], Milo Malanga [3], Jagadeesh Kumar Venkatesan [4], Carmen Alvarez-Lorenzo [5], Magali Cucchiarini [4], Anna Piperno [2,*] and Antonino Mazzaglia [1,*]

1 CNR-ISMN, Istituto per lo Studio dei Materiali Nanostrutturati, V. le F. Stagno d'Alcontres 31, 98166 Messina, Italy; annalaura.cordaro@ismn.cnr.it (A.C); roberto.zagami@ismn.cnr.it (R. Z.)

2 Dipartimento di Scienze Chimiche, Biologiche, Farmaceutiche ed Ambientali, Università di Messina, V. le F. Stagno d'Alcontres 31, 98166 Messina, Italy

3 CycloLab, Illatos út 7, Budapest, Hungary; malanga@cyclolab.hu

4 Center of Experimental Orthopaedics, Saarland University Medical Center, Kirrbergerstr. Bldg 37, Homburg/Saar, D-66421 Germany; jegadish.venki@gmail.com (J.K.V.) mmcucchiarini@hotmail.com (M.C.)

5 Departamento de Farmacología, Farmacia y Tecnología Farmacéutica, I+DFarma (GI-1645), Facultad de Farmacia and Health Research Institute of Santiago de Compostela (IDIS), Universidade de Santiago de Compostela, 15872, Santiago de Compostela, Spain; carmen.alvarez.lorenzo@usc.es (C.A.L.)

* Correspondence: apiperno@unime.it (A.P.); antonino.mazzaglia@cnr.it (A.M.)

1. PolyCD

We estimated 15 CD cavities on the polymer network (PolyCD, Average MW = 25 kDa, CD content ~70%) and 0.21 mmol/g of amino groups. The amount of amino groups was spectroscopically estimated by the colorimetric Kaiser Test [1,2], according to the following equation:

$$NH_2\ loading\ (mmol/g) = \frac{[Abs_{sample} - Abs_{blank}]\ x\ dilution\ (mL)\ x\ 10^3}{\varepsilon\ (M^{-1}cm^{-1})\ x\ sample\ weight\ (mg) x\ optical\ path\ (cm)} \quad (1)$$

where Abs_{sample} is the absorbance at absorption maximum due to the amino groups of the PolyCD sample (λ_{max} = 574 nm), Abs_{blank} was measured at the same conditions but without PolyCD. Dilution was fixed to 5 mL and extinction coefficient (ε) at 574 nm was 15,000 M^{-1} cm^{-1}. Optical path was 0.5 cm.

2 Analysis of release kinetic

The kinetic analysis (Table 3, main text) was performed using three different models proposed in the literature by Higuchi, Baker-Lonsdale and a simple first order process (described in the main text). The first model describes drug release from the matrix as a square root of a time dependent process based on Fickian diffusion. It is possible to resume the Higuchi model with the following expression (generally known as the simplified Higuchi model):

$$C_t = k_H\, t^{\frac{1}{2}} \quad (2)$$

where C_t is the amount of the drug released (in percentage) as function of time t and k_H is the Higuchi dissolution constant.

The Baker-Lonsdale model describes the drug controlled release from a spherical matrix, as follows:

$$\frac{3}{2}\left[1 - \left(1 - \frac{C_t}{C_\infty}\right)^{2/3}\right] - \frac{C_t}{C_\infty} = kt \quad (3)$$

where C_t and $C_\infty$are the amounts of drug released in the receiving phase (in percentage) at time t and t_∞, respectively.

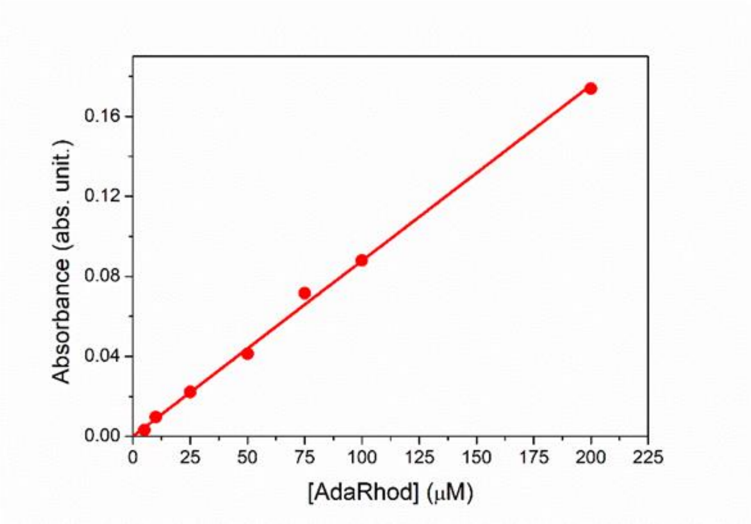

Figure S1. Determination of extinction coefficient by Lambert-Beer law of free Ada-Rhod in DCM at r.t.: $\varepsilon_{(\text{Ada-Rhod } (\lambda \text{max=558 nm}))}$ = 877.8 ± 10 M^{-1} cm^{-1}, (R^2 = 0.99).

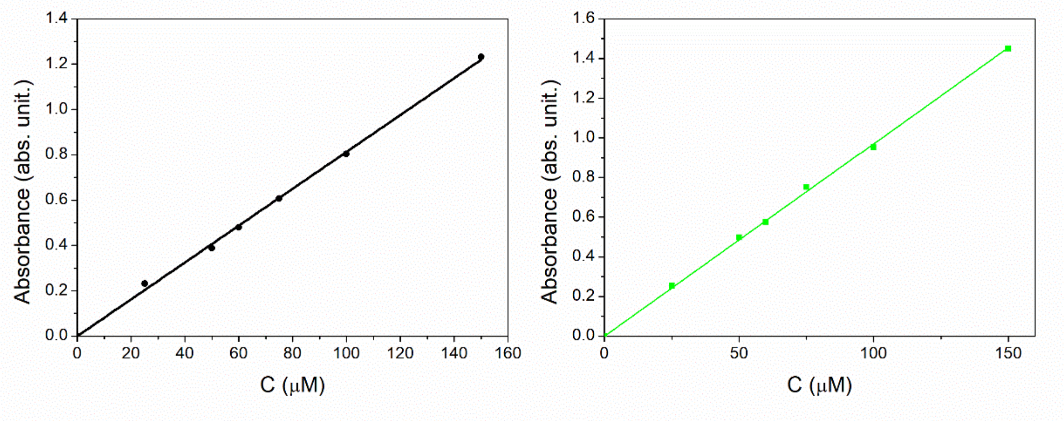

Figure S2. Determination of extinction coefficients by Lambert-Beer law: **A**) in ultrapure water (free DCF, black trace) at r.t.: $\varepsilon_{\text{DCF } (\lambda \text{max=276 nm})}$ = 8130 ± 800 M^{-1} cm^{-1} (R^2 = 0.99); **B**) in PBS (free DCF, green trace) at r.t. : $\varepsilon_{\text{DCF(PBS) } (\lambda \text{max=276 nm})}$ = 9700 ± 767 M^{-1} cm^{-1} (R^2=0.99).

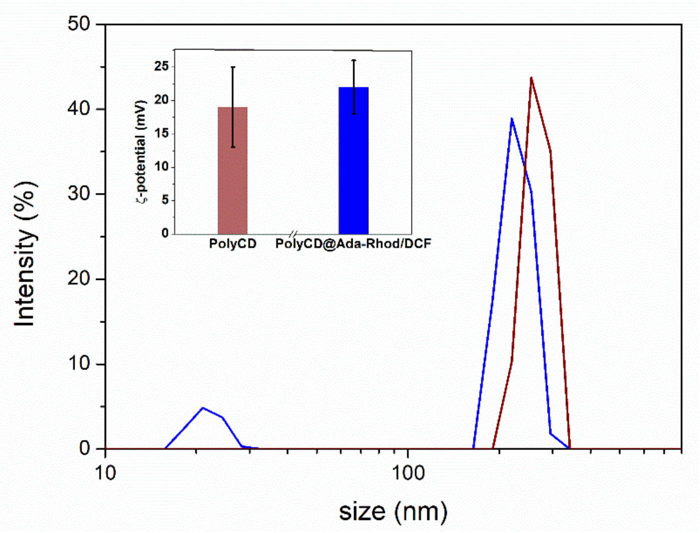

Figure S3. Size (or D_H) distribution of PolyCD (wine trace) and PolyCD@Ada-Rhod/DCF (blue trace) in ultrapure water. In the inset ζ potential ± SD of PolyCD (wine bar) and PolyCD@Ada-Rhod/DCF (blue bar). Experimental conditions: PolyCD and PolyCD@Ada-Rhod/DCF 0.5 mg/mL [DCF] = 236 μM ; [Ada-Rhod] = 8 μM, in ultrapure water at r.t.

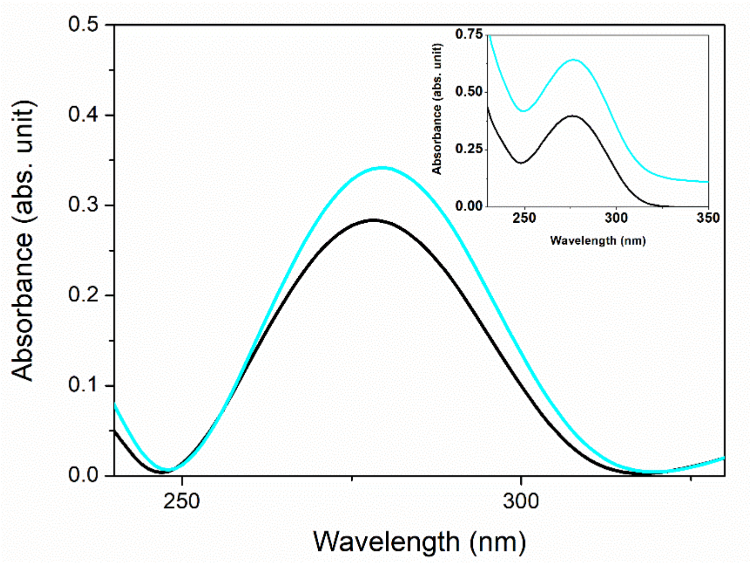

Figure S4. UV/Vis of free DCF (black trace) and PolyCD/DCF (cyan trace) in ultrapure water (scattering was subtracted) obtained by solvent evaporation technique. In the inset, spectra UV/Vis are reported without scattering subctraction. Experimental conditions: [CD] = [DCF] = 35 μM; d = 1 cm, T = 25 °C.

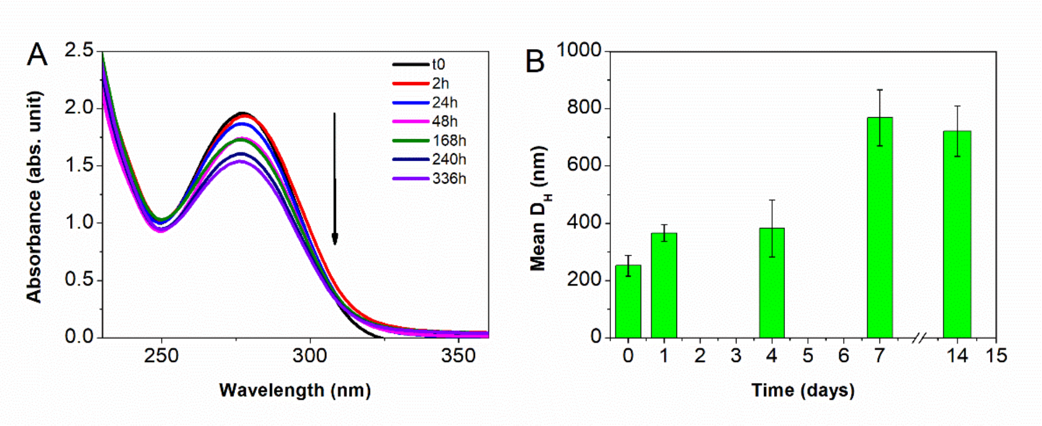

Figure S5. UV spectra (**A**) and mean DH (**B**) (main populations only) vs time of PolyCD@Ada-Rhod/DCF nanoassemblies in NaCl wt. 0.9% (0.5 mg/mL, [Ada-Rhod] = 8 μM, [DCF] = 236 μM). Data were acquired at t = 0, 1, 4, 7 and 14 days. Nanoassembly dispersions were stored at 25 °C along the experimental time.

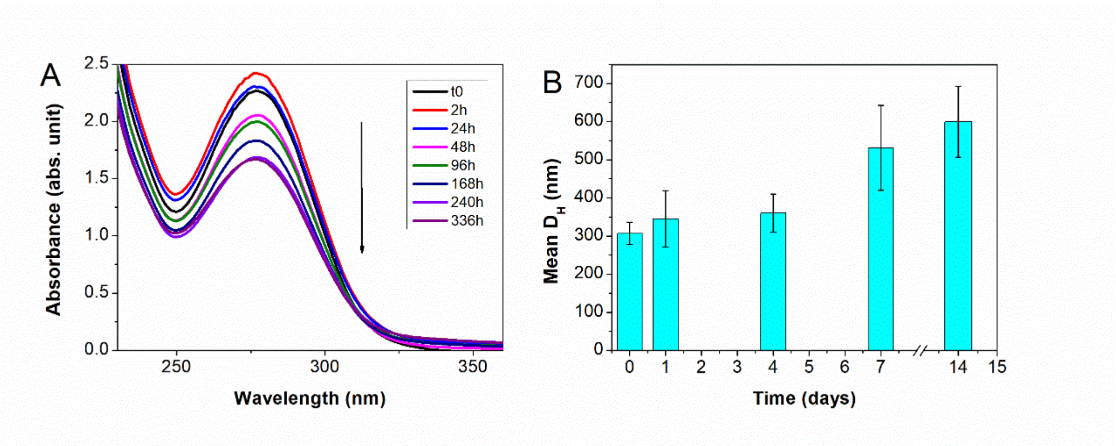

Figure S6. UV spectra (**A**) and mean DH (**B**) (main populations only) vs time of PolyCD@Ada-Rhod/DCF nanoassemblies in in PBS (0.5 mg/mL, [Ada-Rhod] = 8 μM, [DCF] = 236 μM). Data were acquired at t = 0, 1, 4, 7 and 14 days. Nanoassembly dispersions were stored at 25 °C along the experimental time.

References

1. Tuci, G.; Vinattieri, C.; Luconi, L.; Ceppatelli, M.; Cicchi, S.; Brandi, A.; Filippi, J.; Melucci, M.; Giambastiani, G. "Click" on tubes: a versatile approach towards multimodal functionalization of SWCNTs. *Chemistry* **2012**, *18*, 8454-8463, doi:10.1002/chem.201200650.
2. Kaiser, E.; Colescott, R.L.; Bossinger, C.D.; Cook, P.I. Color test for detection of free terminal amino groups in the solid-phase synthesis of peptides. *Analytical biochemistry* **1970**, *34*, 595-598, doi:10.1016/0003-2697(70)90146-6.
